# Effects of salinity acclimation on histological characteristics and miRNA expression profiles of scales in juvenile rainbow trout (*Oncorhynchus mykiss*)

**DOI:** 10.1186/s12864-022-08531-7

**Published:** 2022-04-12

**Authors:** Qi Ling Zhou, Liu Yong Wang, Xiao Long Zhao, Yun Sheng Yang, Qian Ma, Gang Chen

**Affiliations:** 1 College of Fisheries, Guangdong Ocean University, Zhanjiang, 524025 China; 2 Southern Marine Science and Engineering Guangdong Laboratory (Zhanjiang), Zhanjiang, 524025 China

**Keywords:** *Oncorhynchus mykiss*, Scale, Salinity acclimation, Bone homeostasis, microRNA, Histology

## Abstract

**Background:**

The scales serve as an ideal model for studying the regulatory mechanism of bone homeostasis in fish. To explore the effect of salinity acclimation on bone metabolism of juvenile rainbow trout (*Oncorhynchus mykiss*), three sampling time points during salinity acclimation (7D, 14D and 21D) were selected to detect variations in histological characteristics. In the histological analysis, osteoblast marker enzymes alkaline phosphatase (ALP), osteoclast marker tartrate-resistant acid phosphatase (TRAcP) and calcium salt deposit areas (Von Kossa′s) were detected. Changes in calcium (Ca), phosphorus (P) and the molar mass ratio of calcium to phosphorus (Ca/P) in the scales were also detected by Inductively Coupled Plasma Mass Spectrometry (ICP-MS). In addition, the global MicroRNA (miRNA) expression profiles during salinity acclimation were examined using Illumina sequencing platform because of their important regulatory roles in teleost biological processes.

**Results:**

Twelve independent miRNA libraries were constructed, a total of 664 known and 92 putative novel miRNAs were identified. A total of 290 differentially expressed (DE) miRNAs were found in clusters with significant trends in the cluster analysis, and five types of clustering patterns were obtained; 22,374 DE predicted target genes of the aforementioned 290 DE miRNAs were obtained, 5957 of which clustered in six types of clustering patterns with a significant trend. To better understand the functions of the DE miRNAs, GO and KEGG analysis was performed on the 5957 target genes, as a result, they were significantly enriched in bone metabolism related signaling pathways such as MAPK signaling pathway, Calcium signaling pathway, Wnt signaling pathway, Mineral absorption and NF-kappa B signaling pathway. Six DE miRNAs were randomly selected and their expression were verified by quantitative real-time PCR (qRT-PCR), the expression trends were consistent with the results of transcriptome sequencing.

**Conclusions:**

The DE miRNAs and DE target genes identified in this study might play an important role in regulation of bone metabolism during salinity acclimation, relative genes or pathways could serve as key candidates for further studies to elucidate molecular mechanism of teleost bone metabolism, and help performing salinity acclimation and developing marine culture of salmonid species.

**Supplementary Information:**

The online version contains supplementary material available at 10.1186/s12864-022-08531-7.

## Background

Rainbow trout (*Oncorhynchus mykiss*) is a landlocked, cold-water salmonid species, and has long been considered as one of the excellent breeding species worldwide [1]. Similar to other salmonid species such as steelhead trout (*salmon gairdneri*), *O. mykiss* can develop and grow normally in seawater [2]. The strong ability of salinity adaptation makes it possible for these species to be cultured in diving cages in the open sea [3, 4]. The advantage of salinity acclimation has long been focused since it is closely related to the development, quality and timing of transfer of the fish into seawater for aquaculture [5, 6]. Despite the efforts to understand how *O. mykiss* cope with different salinities (osmoregulation mechanism), not much consideration has been given to how the other systems (such as skeletal system) might respond to the salinity adaptation.

Fish skeleton (bones and cartilage) is intimately linked to muscle growth, and is essential for multiple physiological functions such as development, locomotion and load bearing [7]. Similar to the other vertebrates, fish skeleton represents a reservoir of ions such as calcium (Ca), phosphorus (P), and etc., which are in a state of continual exchange with electrolytes found in blood and extracellular fluids [8]. As a complex metabolically active tissue, fish bones undergo continuous remodeling throughout their life. More importantly, bone also plays an important role in plasma ion homeostasis. Previous researches have also demonstrated the importance of linking physiology to biomineral processes [9, 10]. However, the potentially impact of salinity change on bone homeostasis in *O. mykiss* is unclear.

Morphologically, fish bones consist of internal skeleton and exoskeleton. As one of the most important exoskeletons, scales are aligned in partly overlapping rows to protect the fish body. As reported, the scales and skin serve as a barrier to water and ion movements [11]. The removal of scales from just 10% of the body surface would result in disturbing plasma osmolality and sodium levels in juveniles of some teleost species such as *Salmo salar* and *Oncorhynchus tshawytscha* [12, 13]. In addition, scales are easily accessible dermal bone plates that may be useful models in bone research [14]. As reported, calcium management for the creation of new bony materials and the reorganisation and remobilization of Ca^2+^ is present in scales [15]. In other words, a remobilisation of Ca^2+^ can be found in scales and other bone-like structures. As for *O. mykiss*, scales serve as the first source of Ca^2+^ remobilisation followed by fins and bony tissue [16]. This indicates that scales may be the best choice to analyze the regulatory mechanism of bone homeostasis in this species. In addition, resorption of teleost scales has been suggested to be initiated under various physiological and experimental conditions [17]. Considerable interest underlying this subject is to establish the molecular mechanisms of the salinity response of bone homeostasis in fish. However, the aforementioned subjects have not been well studied previously in teleost species.

Normally, the bone metabolism is referred as the modeling and remodeling processes (i.e., the actions of osteocytes, osteoclasts, and osteoblasts) in regulating calcium homeostasis [18]. The influence of salinity on the physiology of bone could be analyzed by measuring the enzymatic activity of alkaline phosphatase (ALP) and tartrate-resistant acid phosphatase (TRAcP), which could respectively represent the osteoblast and osteoclast activity [19–21]. In addition, Ca content could also reflect calcium homeostasis of bone. As reported, precipitation of calcium phosphates was found to be restricted to the outer (episquamal) layer of the elasmoid scale and could be presented by Von Kossa′s staining [22]. Recently, Inductively Coupled Plasma Mass Spectrometry (ICP-MS) has also been used to detect the content of Ca and P in scales [22, 23]. To assess whether bone homeostasis in *O. mykiss* scales is affected by salinity changes, the present study examines the effects of a long-term (21-day) exposure of juvenile *O. mykiss* to sea water, with a specific focus on the impact of salinity on scale miRNA expression profiles.

MicroRNAs (miRNAs) are small, non-coding RNA molecules that regulate gene expression at the post-transcriptional level [24–26]. To date, numerous miRNAs have been discovered in variety of organisms including fish, and the action of these RNAs (with a length of 18–28 nucleotides) was shown to be related to development, homeostasis and many other biological processes [26]. With the application and development of high-throughput technologies, the identification of miRNAs in fish has been widely reported; the studies of these miRNAs have provided new insights into biology [27–29], genome organization, evolution [30], etc. However, the roles of miRNAs in regulating bone homeostasis to environmental change is still unclear. Here, the global miRNA expression profiles in *O. mykiss* scales were studied using Illumina sequencing platform, the differentially expressed miRNAs (DE miRNAs) were characterized to identify novel Ca regulatory or bone homeostasis factors. The results can provide basic information on the molecular signaling pathways involved in bone homeostasis of teleost, and be an important source of information to clarify whether salinity changes could influence the molecular response of bone homeostasis in *O. mykiss*.

## Results

### Histological analysis

#### TRAcP staining of scales

**Fig. 1:**
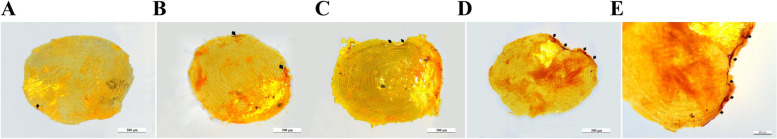
Representative examples of TRAcP staining on the scales of juvenile *O. mykiss* collected at different time points during salinity acclimation. Positive TRAcP staining sites were red (arrow heads), and most of the positive sites were located at the scale margins. (**A**): CG; (**B**): 7D; (**C**): 14D; (**D**): 21D; scale bar = 500 μm, (**E**): 21D; scale bar = 200 μm

The results of TRAcP staining on the scales of juvenile *O. mykiss* were shown in Fig. 1, the positive TRAcP sites were red and mainly located at the scale margins (black arrows). Limited number of positive TRAcP staining sites were identified in the scales of CG, 7D and 14 D; increased number of positive sites was found in the scales of 21 D, and the increased staining was mainly located at the edges of the scales. No resorption pit was identified in scales of all the four groups.

#### ALP staining of scales

**Fig. 2:**
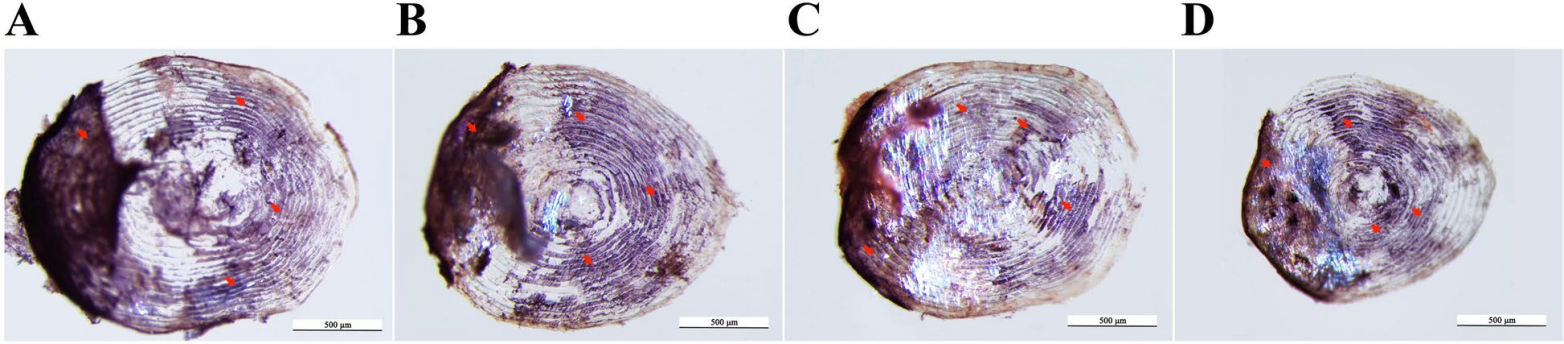
Representative examples of ALP staining on the scales of juvenile *O. mykiss* collected at different time points during salinity acclimation. Positive ALP staining sites were stained purple, and the positive sites were widely distributed on the scales (red arrows). (**A**): CG; (**B**): 7D; (**C**): 14D; (**D**): 21D; scale bar = 500 μm

The results of ALP staining on the scales of juvenile *O. mykiss* were shown in Fig. 2, and the ALP positive sites were stained purple (red arrows). The staining was widely distributed on the scales, and scales of different groups revealed similar ALP distribution.

#### Von Kossa′s staining of scales

**Fig. 3:**
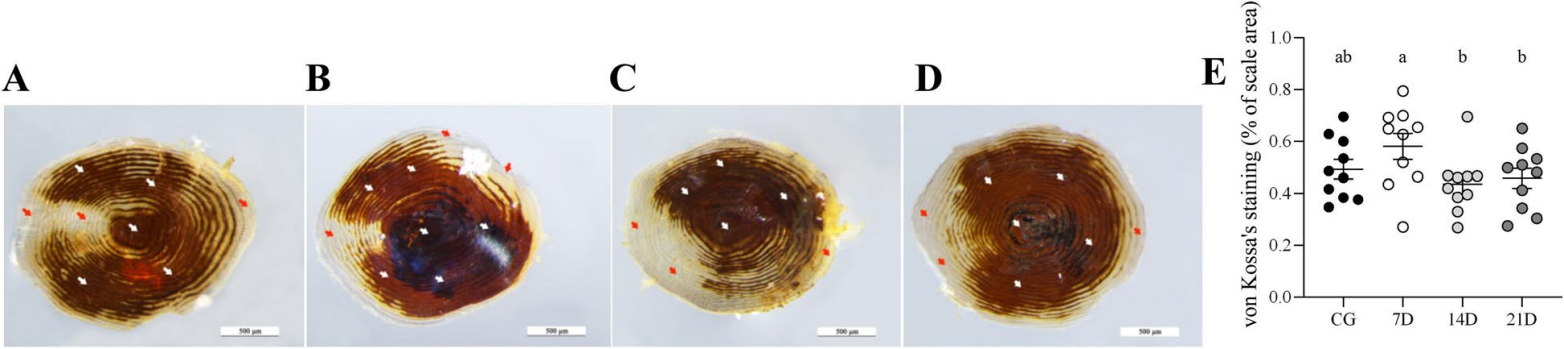
Representative examples of Von Kossa′s staining on the scales of juvenile *O. mykiss* collected at different time points during salinity acclimation, and histomorphometric analyses of the stained scales. Positive Von Kossa′s staining sites (which stains the presence of calcium phosphates a brown colour) were mainly located in the middle of scales (white arrows); and unmineralized areas were mainly at the scale margins (red arrows). (**A**): CG; (**B**): 7D; (**C**): 14D; (**D**): 21D; scale bar = 500 μm. (E): Histomorphometric analyses. Data were expressed as means ± standard deviations ($\overline{\mathrm{x}}$ ± SD); *N* = 10 scales. Different letters represented significant difference (*P*-value < 0.05)

The results of Von Kossa′s staining on the scales of juvenile *O. mykiss* were shown in Fig. 3, and the positive sites of calcium salts were stained brown. A large area of positive Von Kossa′s staining was identified in the middle of the scales. The positive Von Kossa′s staining in 7D scales was stronger than scales of the other three groups (Fig. 3B and E). The location of Von Kossa′s staining area was similar to that of the ALP staining area, and the calcium salt deposits less in the TRAcP positive staining area.

#### Calcium and phosphorus analysis of scales

**Fig. 4:**
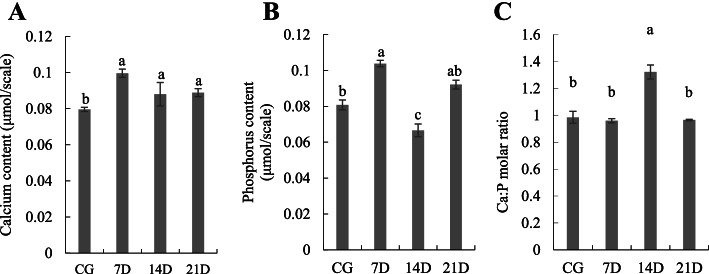
The calcium (Ca), phosphorus (P) and calcium/phosphorus molar mass ratio (Ca/P) in scales of juvenile *O. mykiss* were respectively shown in (**A**), (**B**) and (**C**)*.* CG represented freshwater control group; 7D, 14D and 21D respectively represented seawater acclimation of seven, 14 and 21 days. Data were expressed as means ± standard deviations ($\overline{\mathrm{x}}$ ± SD); *N* = 24 scales. Different letters represented significant difference (*P*-value < 0.05)

Changes in Ca content of the scales throughout the experimental period were shown in Fig. 4(A), and the result was consistent with the Von Kossa′s staining results. The Ca content first increased and reached the highest level (0.100 μmol/scale) at 7D. The Ca content of the scales at 7D, 14D and 21D were significantly higher than that of the CG scales (*P*-value < 0.05). The P content of the scales [Fig. 4(B)] also reached the highest level (0.104 μmol/scale) at 7D, which was significantly higher than that of the CG scales (0.081 μmol/scale) (*P*-value < 0.05); the 14D scales exhibited the lowest P content (0.067 μmol/scale) (*P*-value < 0.05).

The molar mass ratio of calcium to phosphorus (Ca/P), indicating the crystalline phase of calcium phosphate (e.g., pure calcium hydroxyapatite, theoretical ratio = 1.67), was also shown in Fig. 4(C). The Ca/P of 14D scales was significantly higher than scales of the other three groups, and no significant difference was found among Ca/P of CG, 7D and 21D scales.

#### Illumina sequencing of miRNAs in *O. mykiss*

To identify miRNAs in the scales of juvenile *O. mykiss*, twelve miRNA libraries from CG (CG_1, CG_2 and CG_3), 7D (7D_1, 7D_2 and 7D_3), 14D (14D_1, 14D_2 and 14D_3) and 21D (21D_1, 21D_2 and 21D_3) were respectively constructed and sequenced using Illumina sequencing technology. A total number of 11,277,023, 11,052,110, 12,969,211 and 11,929,493 raw reads were respectively obtained from CG, 7D, 14D and 21D scales. After preprocessing steps, a total of 921,415, 824,625, 946,666 and 894,351 clean reads were obtained due to the removal of low-quality reads, adapters, etc. The aforementioned reads respectively represented 341,328, 261,252, 245,627 and 236,173 unique sequences (valid reads) (Table S1). Furthermore, the length distribution analysis of these miRNA sequences showed a similar pattern of distribution in length of all libraries (Fig. S1). The length of the miRNAs in all libraries varied from 18 to 26 nt and the majority of read length was 22 and 23 nt, followed by 21, 24, 25 and 26 nt. The read length of 22 nt in the four groups respectively accounted for 23.31, 24.75, 33.40 and 35.18% of the total miRNA numbers. The raw reads of the twelve libraries were uploaded into the NCBI database Sequence Read Archive (SRA) and the SRR numbers were from SRR15559621 to SRR15559632.

#### Identification and specific expression of miRNAs in *O. mykiss* scales

To identify miRNAs in the scales of juvenile *O. mykiss*, all valid sequences were compared with known vertebrate miRNAs and miRNA precursor sequences in miRBase database. As a result, a total of 756 (664 known and 92 putative novel miRNAs) miRNAs were identified (Table S2). These miRNAs covered 121 miRNA families, and the most abundant families were let-7 (40 members), miR-10 (29 members) and miR-30 (24 members) (Table S3).

**Fig. 5:**
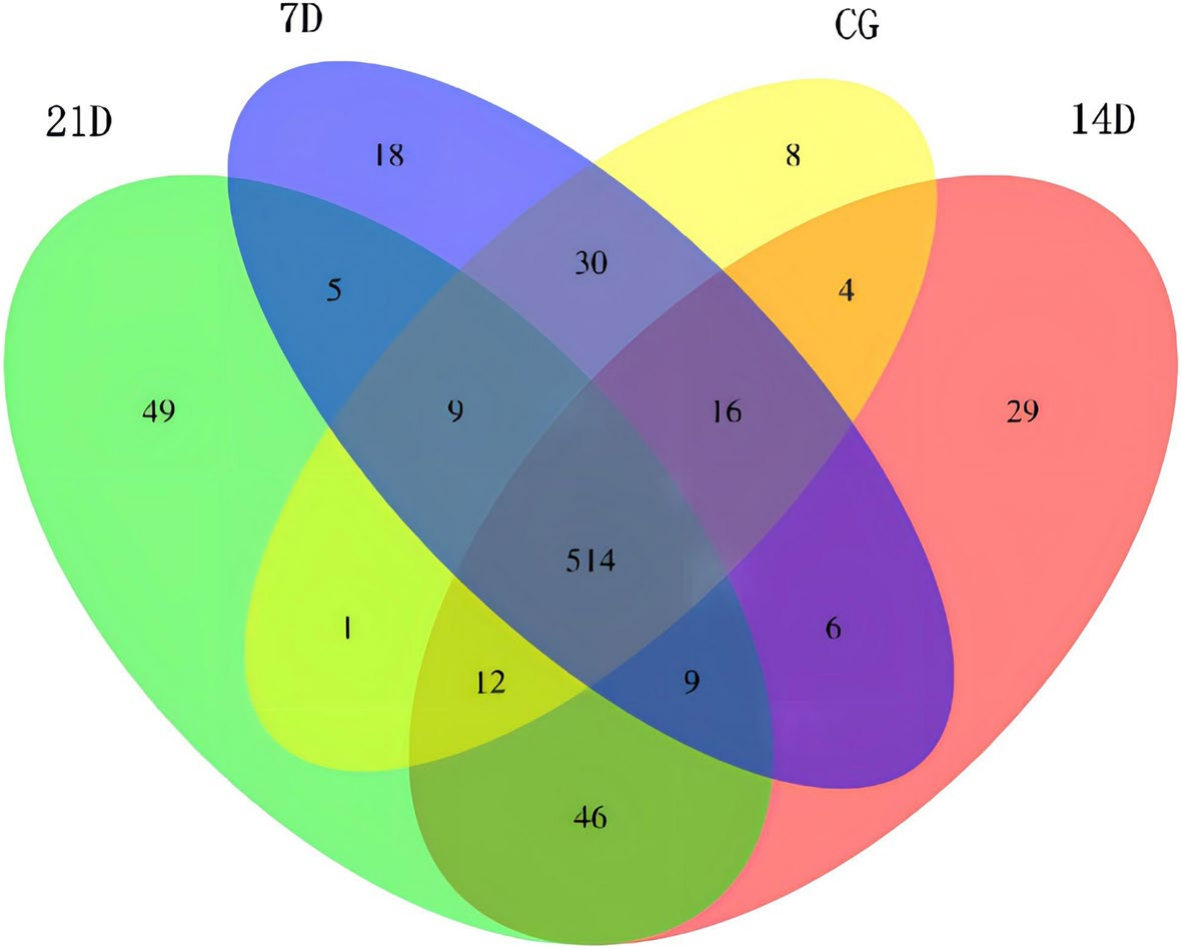
Venn diagram showing the number distribution of miRNAs expressed in *O. mykiss* scales collected at different time points during salinity acclimation

In the 756 miRNAs, 514 miRNAs showed co-expressed in all the four groups (Fig. 5). Eight,18, 29 and 49 miRNAs were uniquely expressed in CG, 7D, 14D and 21D scales, respectively. In all the four groups, 21D scales exhibited the largest number of unique miRNAs. 594 miRNAs were expressed in scales of CG, 607 were expressed in 7D, 636 were expressed in 14D, and 645 were expressed in 21D.

#### Transcriptome sequencing data

RNA-Seq was performed in juvenile *O. mykiss* scales of each sample, and a total of 147.29 Gb valid data was obtained. Quality of the RNA-Seq data and comparison of mRNA sequences from different groups were shown in Table 2. The valid data of each sample reached 11.22 Gb, and the minimum percentage of Q30 bases was 97.32%. The valid data of each sample was compared with the *O. mykiss* genome, and the minimum comparison efficiency reached 82.74%.

#### Differential expression analysis and target gene prediction of the identified miRNAs

**Fig. 6:**
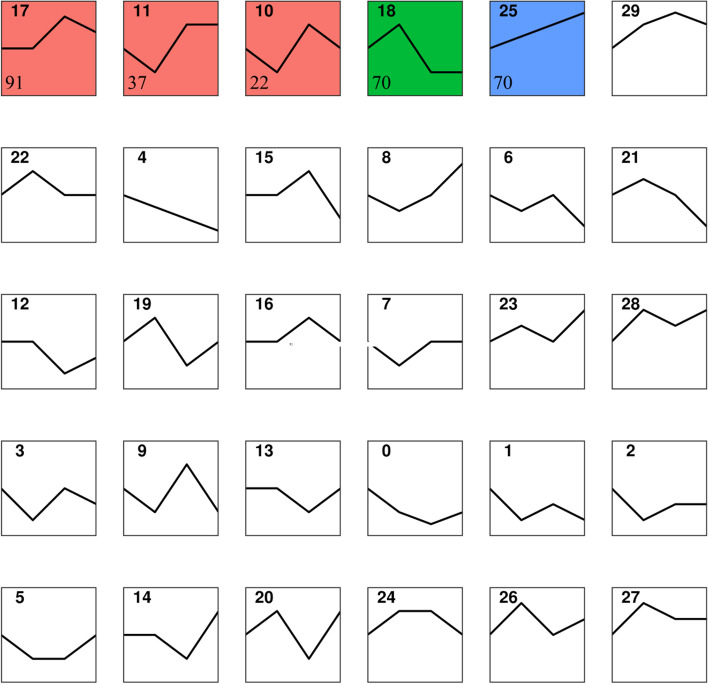
Sketchmap of the cluster analysis of DE miRNAs in scales of juvenile *O. mykiss* collected at different time points during salinity acclimation. Filled color in the clusters indicated significant trends (*P*-value < 0.05). The number in the top left corner represents the type order. The number in the lower left corner represents the number of miRNAs in the cluster

The trend of miRNA expression at different timepoints (7D, 14D and 21D) was analyzed using STEM software. As shown in Fig. 6, the 327 DE miRNAs were clustered into 30 expression patterns, of which five were with significant changing trends (Cluster 10, Cluster 11, Cluster 17, Cluster 18 and Cluster 25) (*P*-value < 0.05). A total of 290 CST miRNAs (DE miRNAs from clusters with significant trends in the cluster analysis, CST miRNAs) were clustered into these five patterns (contains 22 CST miRNAs in Cluster10; Cluster 11 contains 37 CST miRNAs; Cluster 17 contains 91 CST miRNAs; Cluster 18 contains 70 CST miRNAs; Cluster 25 contains 70 CST miRNAs), detailed information was listed in Table S4. A total of 22,374 predicted target genes of the CST miRNAs were obtained from the aforementioned five expression modes; the CST miRNAs in Cluster 10, Cluster 11, Cluster 17, Cluster 18 and Cluster 25 each had 3897, 5756, 9423, 8586 and 8977 predicted target genes.

**Fig. 7:**
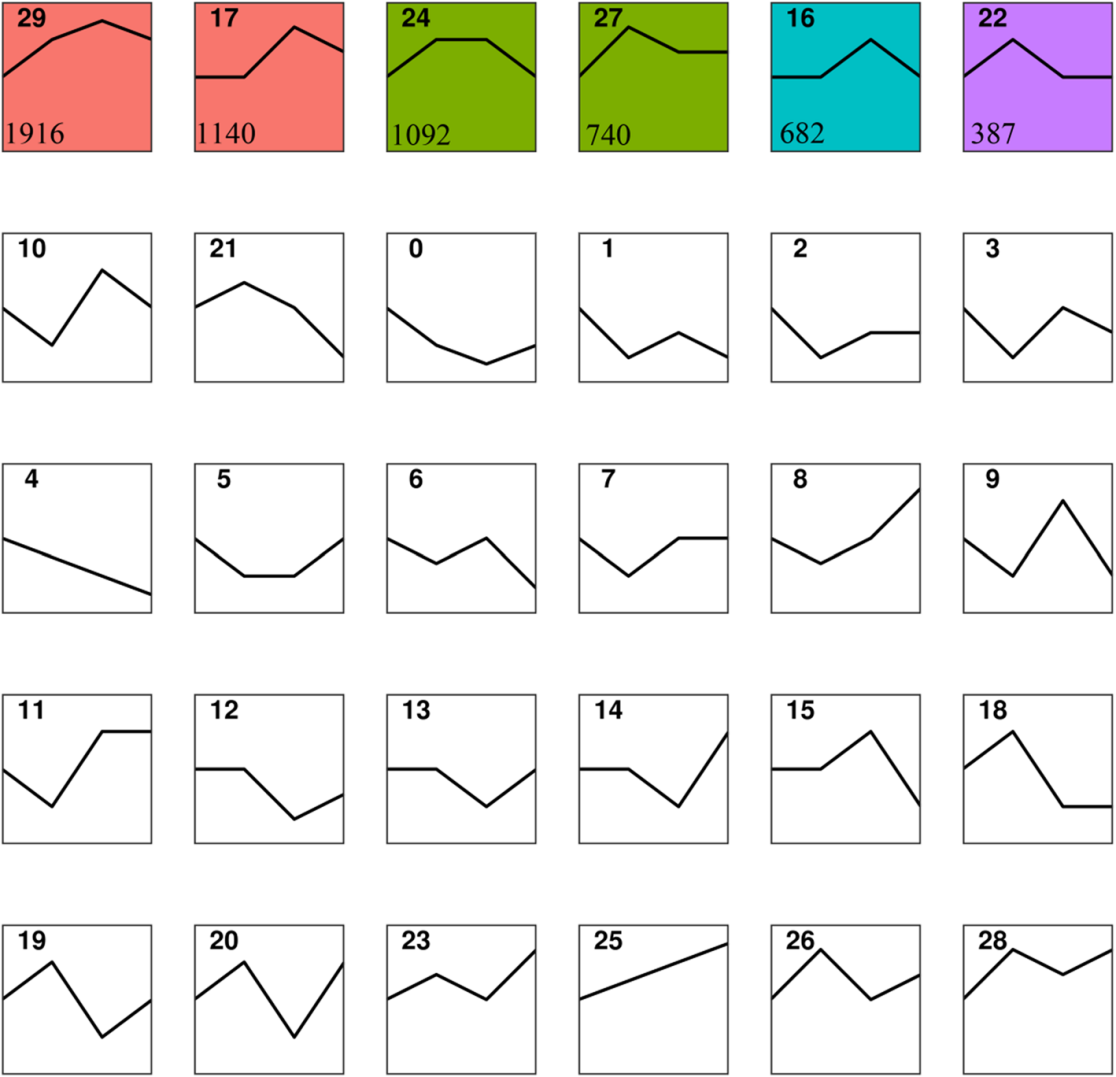
Sketchmap of the cluster analysis of DE mRNAs in scales of juvenile *O. mykiss* collected at different time points during salinity acclimation. Filled color in the clusters indicated significant trends (*P*-value < 0.05). The number in the top left corner represents the type order. The number in the lower left corner represents the number of target genes in the cluster

Additionally, trend analysis was performed on the 22,374 predicted target genes of CST miRNAs. The co-clustering was also divided into 30 expression patterns, among which 5957 CST mRNAs (DE mRNAs from target genes of 22,374 CST miRNAs showing a significant trend in cluster analysis, CST mRNAs) were clustered into six expression patterns with significant changing trends (Cluster 16, Cluster 17, Cluster 22, Cluster 24, Cluster 27 and Cluster 29) (*P*-value < 0.05) (Fig. 7). Detailed information was listed in Table S5. Cluster 16 contains 682 CST mRNAs; Cluster 17 contains 1140 CST mRNAs; Cluster 22 contains 387 CST mRNAs; Cluster 24 contains 1092 CST mRNAs; Cluster 27 contains 740 CST mRNAs; Cluster 29 contains 1916 CST mRNAs.

#### GO analysis of CST mRNAs

**Fig. 8:**
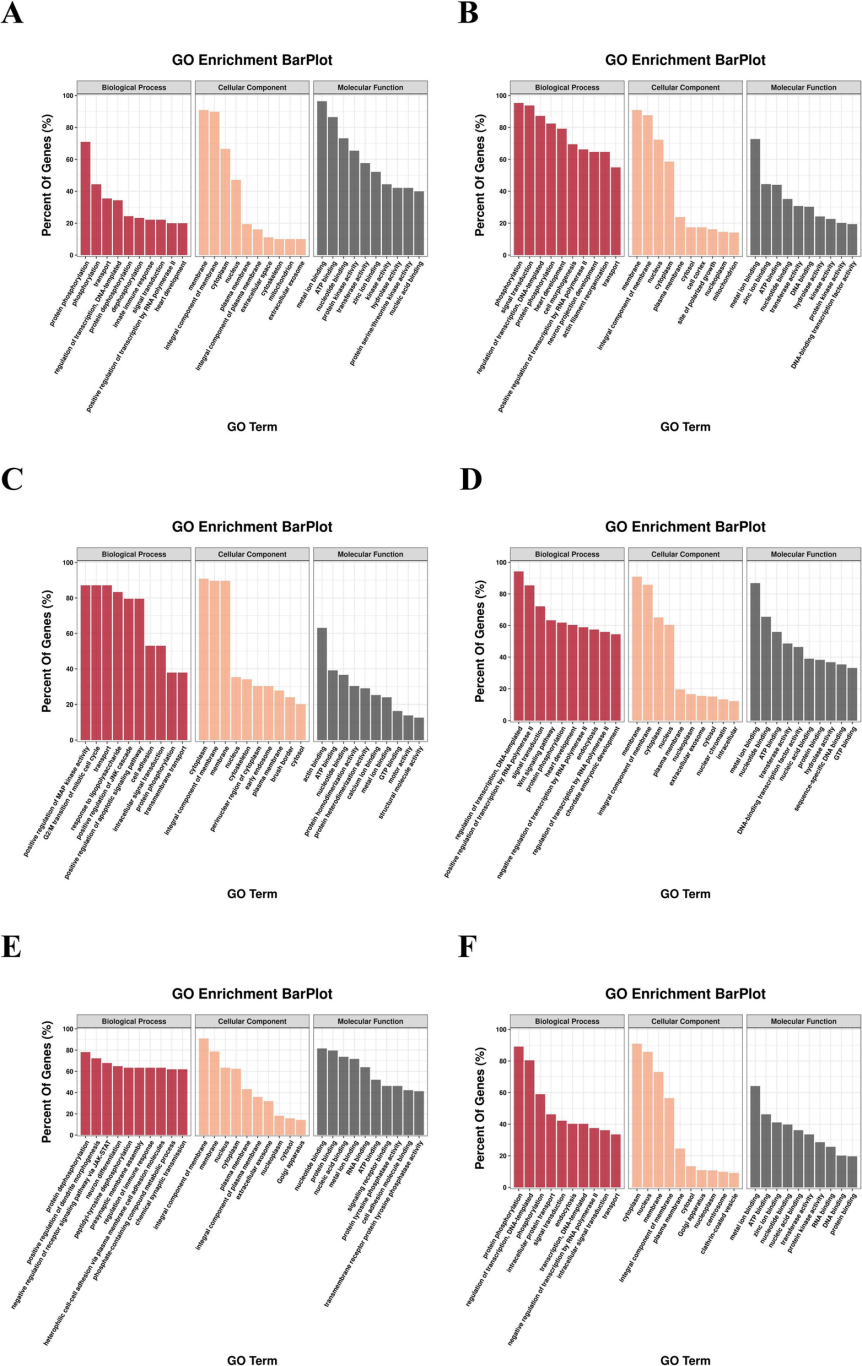
Gene ontology (GO) annotation of CST mRNAs in scales of juvenile *O. mykiss* collected at different time points during salinity acclimation. Each color represents a categorie. **A**: Cluster 16; **B**: Cluster 17; **C**: Cluster 22; **D**: Cluster 24; **E**: Cluster 27; **F**: Cluster 29. (According to the Fig. 7)

Gene ontology (GO) annotation (function) analysis was performed on predicted CST mRNAs to explain and speculate the function of CST mRNAs (Fig. 8). Detailed information of the GO analysis was listed in Table S6. As shown in Fig. 8, the predicted CST mRNAs could be enriched in different GO terms such as biological process (BP), and cellular component (CC) and molecular function (MF). The columns in each category presented in the figure were in descending order of percent of genes. The CST mRNAs in the BP category were enriched in regulation of transcription, DNA-templated, phosphorylation, signal transduction, transport, etc. The CST mRNAs in the CC category were associated with terms such as membrane, integral component of membrane, cytoplasm, nucleus and cytosol. In the MF category, the CST mRNAs were associated with terms such as metal ion binding, ATP binding, nucleotide binding and transferase activity.

#### KEGG pathway analysis of CST mRNAs

**Fig. 9:**
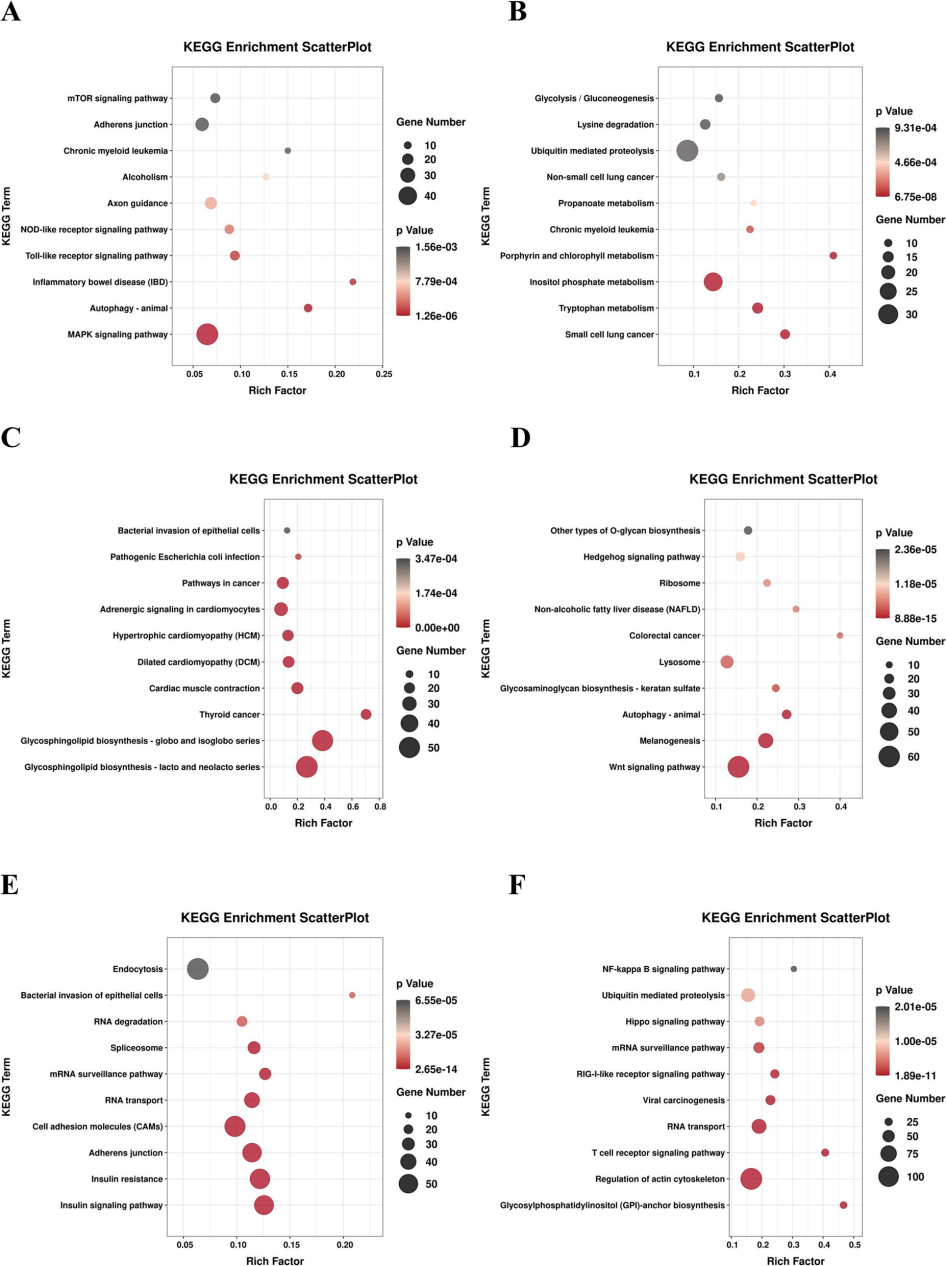
Pathway classifications of CST mRNAs according to KEGG results of juvenile *O. mykiss* scales collected at different time points during salinity acclimation. Size of circle represented size of enriched CST mRNAs number. Shade of color represented size of *P*-value. (**A**): Cluster 16; (**B**): Cluster 17; (**C**): Cluster 22; (**D**): Cluster 24; (**E**): Cluster 27; (**F**): Cluster 29. (According to the Fig. 7)

Kyoto Encyclopedia of Genes and Genomes (KEGG) analysis was performed to analyze the biological pathways of the predicted CST mRNAs (Fig. 9). Detailed information of the KEGG pathways (*P*-value < 0.05) was listed in Table S7. predicted CST mRNAs were significantly enriched in the pathways such as the MAPK signaling pathway, Toll-like receptor signaling pathway, mTOR signaling pathway, Thyroid hormone signaling pathway, Calcium signaling pathway, VEGF signaling pathway, ErbB signaling pathway, Insulin secretion, Wnt signaling pathway, TGF-beta signaling pathway, FoxO signaling pathway, Insulin signaling pathway, Mineral absorption, NF-kappa B (NF-κB) signaling pathway and other bone metabolism related signaling pathways.

#### Validation of DE miRNAs using qRT-PCR

**Fig. 10:**
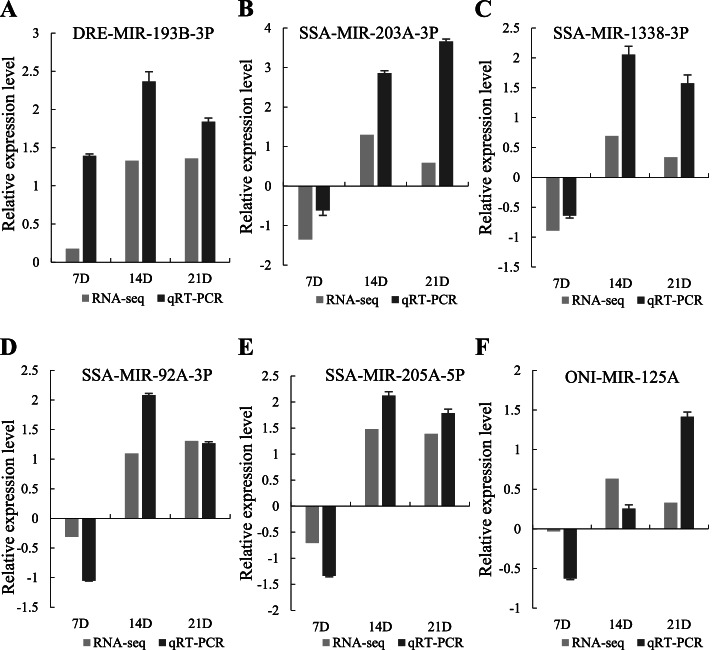
qRT-PCR verification of DE miRNAs. The relative expression levels of six miRNAs at 7D, 14D and 21D were calculated according to log_2_(fold change) and based on the control group (CG) expression level of zero, expressed as means ± standard deviations ($\overline{\mathrm{x}}$ ± SD, *N* = 3). Six miRNAs were significantly different from CG at 7D, 14D and 21D (*P*-value < 0.05)

A total of six randomly selected miRNAs, including DRE-MIR-193B-3P, SSA-MIR-203A-3P, SSA-MIR-1338-3P, SSA-MIR-92A-3P, SSA-MIR-205A-5P and ONI-MIR-125A, were used for qRT-PCR analysis. As shown in Fig. 10, the expression pattern of the six miRNAs were consistent with the transcriptome sequencing results.

## Discussion

Based on the TRAcP staining results of scales in juvenile *O. mykiss*, positive sites were mainly found along the edges of the scales. This location was similar to that of the zebrafish scales, which showed a detection of TRAcP along the entire length of the grooves and in the focus. Previously, the TRAcP has been used as a marker for indicating osteoclastic activity in *O. mykiss* scales, and it has been reported that the TRAcP activity could be induced by injection of estradiol-17β [23, 31]. Here in this study, the TRAcP activity in the scales was increased at 21D, which revealed an potential effect of salinity acclimation on the osteoclast activity. In addition, scales of the four groups revealed similar ALP staining patterns. According to these results, an increase on the absorption of calcium in the scales was anticipated because that the activation of osteoclast always resulted in demineralization. The calcium salt deposition of scales was first increased at 7D and then followed by a decrement along with the salinity acclimation based on the Von Kossa′s staining and ICP-MS results. The possible reasons were analyzed as followed: (1) juvenile *O. mykiss* were used for the salinity acclimation, skeleton of fish at the early developmental stages were in the period of rapid growth, and high osteoblast activity was maintained. The effect of salinity acclimation on osteoclasts was not strong enough to exceed the effects of the high osteoblast activity. (2) demineralization is a relatively slower process than the changes in enzymes or cell activity. Even though salinity acclimation stimulated the absorption of exogenous calcium, it might take time for the unmineralization to process.

As for the transcriptome sequencing, a total of 756 (664 known and 92 putative novel miRNAs) miRNAs and 290 CST miRNAs were identified in *O. mykiss* scales collected along with the salinity acclimation. The number of the identified miRNA were larger than those of the skeleton tissues (bone and cartilage) in mammal species. For instance, Desjardin [32] identified miRNAs in equine subchondral bone and cartilage by next-generation sequencing, 622 and 609 miRNAs were respectively identified, including about 300 novel miRNAs, and the results showed that they were involved in the physiopathological process of osteochondrosis. As for the articular cartilage of rats, a total of 310 known miRNAs as well as 86 novel miRNAs candidates were identified, these miRNAs were found to be targeting chondrocyte proliferation and differentiation regulatory factors, such as Wnts, BMPs, Runx2, Sox9, HDAC4, etc. [33].

In order to explore the role of DE miRNAs in the regulation of *O. mykiss* bone metabolism, KEGG enrichment analysis was performed on the target genes of these miRNAs. As a result, a few DE miRNAs were screened out because their target genes were mainly enriched in bone metabolism-related signal pathways. As for the bone homeostasis in teleost, both bone resorption (osteoclast formation and differentiation, osteoblast/chondrocyte apoptosis) and bone formation (osteoblast/chondrocyte formation and differentiation, inhibition of osteoclast) are important processes [18]. Here in this study, some of the mentioned DE miRNAs were related to regulation of promoting osteoclast formation and differentiation or chondrocyte apoptosis. Up-regulation of these miRNAs were detected in accordance with the increment of TRAcP staining, indicating the impact of salinity acclimation on bone resorption.

The target genes of DE miRNAs SSA-MIR-30B-5P_R-1, SSA-MIR-181A-5P, GMO-MIR-181B-5P_R-1 and GMO-MIR-199-5P_R + 1 were significantly enriched in the NF-κB signaling pathway. This pathway has been considered to be a common final pathway for many inflammatory mechanisms in the pathophysiology of cartilage [34]. It has been reported that the activation of NF-κB could induce the secretion of cytokines such as IL-1β, IL-18 and TNF-α, which would have an effect on cartilage matrix remodeling, chondrocyte apoptosis, synovial inflammation, and cause cartilage damage [35]. Genes in this pathway could also participate in osteoclast formation, differentiation, maturation and anti-apoptosis of vertebrate [34]. As reported, the target gene of SSA-MIR-181A-5P and GMO-MIR-181B-5P_R-1 such as NF-κB inhibitor alpha-like and calreticulin could activate the NF-κB signaling pathway, and ultimately achieve the purpose of promoting osteoclast formation and bone resorption.

In addition, the DE miRNAs tni-miR-181a-5p and ola-miR-181b-5p_R-1 were found to be targeting tlr2, which was an important factor in the Toll-like receptor (TLR) signaling pathway. TLR2 could induce mouse macrophages to secrete TNF-α or activating NF-κB to promote osteoclast formation and bone resorption [36]. Another important signaling pathway is the MAPK signaling pathway. By promoting the overexpression of IL-1β and TNF-α, activation of MAPK signaling pathway could lead to cartilage damage in articular of Vertebrate [37]. Significant difference in the expression of dre-miR-181a-5p_L + 2 and its target gene IL-1β were identified between salinity acclimation and CG scales in this study. Furthermore, the DE miRNAs gmo-miR-23a-3p, tni-miR-23b and ssa-miR-19c-3p were also found to be targeting TNF. These results indicated that these genes might be involved in the apoptosis of chondrocytes [38].

Among the CST miRNAs, SSA-MIR-26A-5P, ONI-MIR-27B, GMO-MIR-27D-5P_L + 1R-2, GMO-MIR-125B-3P_R + 3, SSA-MIR-133B-3P_L-1R + 2, DRE-MIR-204-5P_R + 2, ONI-MIR-206 and other miRNAs target genes were mainly enriched in Wnt, TGF-β, Notch and other bone metabolism-related signaling pathways. According to Friedlander [39], some genes in the classic Wnt pathway can not only promote the differentiation of mouse osteoblasts, the formation and mineralization of bone matrix. In this study, oni-miR-27e and ssa-miR-29a-3p_R + 2_1ss18AG could enhance Wnt signal transduction by down-regulating the negative regulatory gene dkk1 in the Wnt signaling pathway, thereby promoting osteoblast differentiation. It was consistent with the research results of Westendorf [40] in hFOB culture in vitro. It was speculated that oni-miR-27e and ssa-miR-29a-3p_R + 2_1ss18AG were important osteoblast differentiation regulating miRNAs. In this study, according to GO and KEGG annotation results, it was speculated that GMO-MIR-125B-3P_R + 3 inhibited osteoblast differentiation by targeting bmp-2. Cbfb gene is an essential cofactor for the transcription factor Runx2 during osteoblast differentiation. Huang [41] found that over-expression of miR-125b in C3H10T1/2 cells could interfere with the expression and down-regulate Cbfb protein, and over-expression reduced the mRNA levels of three osteogenic marker genes including ALP, osteocalcin (OCN) and osteopontin (OPN) induced by BMP-2, whereas, anti-mir-125b increased the expression of these marker genes and hence up-regulated mRNA levels of Cbfb. In this study, dre-miR-125c-5p_R + 1_1ss18CT was also found to play a regulatory role in osteoblast differentiation by targeting cbfb. The results showed that miR-125b was a key regulator of osteoblast differentiation.

When comparing the GO analysis results of DE miRNA and their target genes, they were both mainly enriched in blood vessel development, metal ion binding, ATP binding, calcium ion binding and transport, etc., indicating that the identified DE miRNAs responded to salinity acclimation mainly by regulating the target genes related to these pathways. KEGG enrichment analysis of DE miRNAs and their target genes also exhibited a similar result in the significant enriched pathways such as MAPK signaling pathway, Calcium signaling pathway, VEGF signaling pathway, Wnt signaling pathway, NF-κB signaling pathway.

## Conclusion

The effect of salinity acclimation on bone metabolism of *O. mykiss* was confirmed based on the histochemistry and morphometric analysis of the scales in CG, 7D, 14D and 21D, showing that osteoclastic activity and the calcium salt deposition of scales were significantly increased. Then the transcriptome analysis of the aforementioned samples was conducted using RNA-seq. Variations in expression patterns of miRNAs were identified, GO and KEGG pathway analysis of the DE miRNAs revealed that they might play important roles in regulation of bone metabolism during salinity acclimation. Our findings provide candidate key genes or pathways for follow-up work to elucidate molecular mechanism of teleost bone metabolism, which could help performing salinity acclimation and developing marine culture of salmonid species.

## Materials and methods

### Preparation and management of *O. mykiss*

Juveniles of *O. mykiss* (body weight 46.9 ± 7.9 g and body length 15.8 ± 1.4 cm) were collected at Linqu Hatchery Station (Shandong, China) and the experiment was carried out at the same place. A total of 90 fish were equally separated into three groups, each group was domesticated in a flat bottom Fiber Reinforce Plastic (FRP) tank with an effective volume of 1000 L under a 12 h light: 12 h dark photoperiod for 7 days (d) prior to the beginning of the experiment. At the start of the experiment, samples were collected as control group (CG) while fish remained at freshwater 3‰. Salinity change commenced thereafter by adding seawater into the inflowing water to each tank, salinity was gradually increased at a rate of 4‰ per day for a six-day period, and then the fish were maintained in this salinity (27‰) afterwards. Scale samples were collected from three individual fish at the time points of 7 days (7D), 14 days (14D) and 21 days (21D).

### Sample collection

Juvenile *O. mykiss* from each group were randomly selected and euthanized with MS-222 (3-Aminobenzoic acid ethyl ester methanesulfonate), ontogenic scales of each fish were removed with forceps from a 2 cm × 1 cm area on the left flank of the fish (extending from the posterior base of the dorsal fin to the base of the caudal fin) and then immediately frozen in liquid nitrogen. A total of approximately 100 scales per fish were collected, thirty of which were used for histochemistry and morphometric analysis, eight were used for ICP-MS, and the rest (about 60 scales) were used for transcriptome sequencing and quantitative real-time PCR (qRT-PCR).

### Histological analysis

For whole mount studies, scales were fixed in 4% paraformaldehyde in phosphate buffered saline (PBS, pH = 7.8) at 4 °C for 24 h. Subsequently, three different kinds of staining experiments were respectively carried out on the scales of *O. mykiss* from four sample groups (CG, 7D, 14D and 21D). Each group was consisting of three fish in parallel, and six scales of each fish were used in parallel for dyeing. Scales were stained for activity of TRAcP and ALP, according to the instruction of Tartrate-Resistant Acid Phosphata stain Kit (D023–1) and BCIP/NBT stain Kit (I023–1) (Nanjing Jiancheng Bioengineering Institute, Nanjing, China) respectively.

In addition, samples were stained for presence of calcium phosphates using Von Kossa′s Kit (G3282) (Beijing Solarbio Science & Technology Co., Ltd., Beijing, China), following the manufacturer’s instructions. Breifly, scales were incubated with 5% silver nitrate under bright light for 1 h, subsequently rinsed with tap water for 1 min, developed for 5 min in 5% sodium thiosulfate, and finally rinsed thoroughly with tap water. All samples were photographed under a Leica M205 FCA stereo microscope (Leica microsystems, Wetzlar, Germany) and the stained area of Von Kossa′s stained scales was quantified with Photoshop 2019 (Adobe, San Jose, USA).

### Calcium and phosphorus analysis

Eight ontogenetic scales of each fish were washed with ultrapure water, dried and placed in a Teflon container and digested using 6 mL of HCl-HNO3-HF-H2O2 (3:1:1:1) in a microwave oven (Anton Paar Multiwave PRO 41HVT56, Austria) [42]. And then, the Teflon container was placed on a heating block (180 °C) to remove the acid [43]. Quantitatively transfer the digestion solution to a 50 mL colorimetric tube by adding ultrapure water. The residual digested solution in the Teflon container was also transferred by rinsing the wall at least 3 times with ultrapure water. The resulting solution was diluted with ultrapure water and then analyzed, with the resulting solution was corrected with blank acid. ICP-MS (Agilent 7500 Cx, USA) was used to measure the content of Ca and P in the digestion solution of each sample, and calculate Ca/P [44]. Details of quality assurance (QA) and quality control (QC) can be found in Additional file 9 (Supplemental Materials and Methods).

### RNA extraction, library construction, and sequencing

The scale samples were homogenized using mortal/pestle and liquid nitrogen and then used for RNA extraction. Total RNA was respectively isolated from *O. mykiss* scale samples at CG, 7D, 14D and 21D group using TRIzol reagent (Invitrogen, CA, USA) according to the manufacturer’s procedure. The quality of total RNA was analyzed by Bioanalyzer 2100 (Agilent Technologies, Santa Clara, CA, USA), and the concentration and the quality of the total RNA was analyzed using the NanoDrop 2000 (Thermo Fisher Scientific, Lafayette, CO, USA). RIN score ≥ 7 was accepted. And the RNA concentration used for library preparation was ≥200 ng/uL. Twelve small RNA libraries were constructed following the procedure from TruSeq Small RNA Sample Prep Kits (Illumina, San Diego, USA). According to the PrimeScript™ II 1 st strand cDNA synthesis Kit (TaKaRa) instructions, 1 μg total RNA was reversely transcribed into first-strand cDNA. The libraries were then single-end 50 bp sequenced using an Illumina Hiseq 2000/2500 platform (Lc-bio, Hangzhou, China). For the transcriptome sequencing, a chain-specific library was constructed by rRNA depletion. After the library was qualified, Illumina Hiseq 4000 was used for sequencing, and the sequencing read length was 2*150 bp (PE 150).

### Identification of DE miRNAs

The miRNA sequences were identified by ACGT101-miR (LC Sciences, Houston, Texas, USA). In briefly, adapter dimers, low complexity, common RNA families (rRNA, tRNA, snRNA, snoRNA) and repeats in raw reads were removed by cutadapt V1.10 software. Subsequently, unique sequences with a length of 18 ~ 26 nucleotide were mapped to specific species precursors in miRBase 22.0 by BLAST search to identify known miRNAs and novel 3p- and 5p- derived miRNAs. The unmapped sequences were BLASTed against the specific genomes, and the hairpin RNA structures containing sequences were predicated from the flank 80 nt sequences using RNAfold software (http://rna.tbi.univie.ac. at/cgi-bin/RNAfold.cgi). To identify DE transcripts across samples or groups, the edgeR package was used. We identified miRNA with a fold change ≥2 and *P*-value < 0.05 in a comparison as DE miRNAs.

### Prediction and enrichment analysis of genes targeted by miRNAs

To predict target genes (mRNAs) from all known and novel CST miRNAs, two computational target prediction algorithms (TargetScan 50 and Miranda 3.3a) were used to identify miRNA binding sites. And the parameters of TargetScan were set as more than or equal to 90, the parameters of Miranda were set as less than − 20 kcal/mol. Finally, the data predicted by both algorithms were combined and the overlaps were calculated. GO annotation and KEGG pathway enrichment were performed to determine the possible functions of DE miRNA and DE mRNA by mapping them to GO (http://www.geneontology.org/) and KEGG (http://www.genome.jp/kegg/) databases the thresholds were set as *P*-value < 0.05.

### Express trend analysis of miRNAs and their target genes

Short Time-series Expression Miner (STEM, version1.2.2b) software was used to perform trend cluster analysis of the miRNAs and target genes expression patterns in four groups.

### Transcriptome sequencing and analysis

Using the cutadapt V1.10 software, the joint reads and the low-quality data reads in the raw data were removed to obtain the valid data reads. The obtained reads were assembled and quantified using the transcript assembly software StringTie 1.3.0. Then, the expression level for mRNAs were performed by calculating fragments per kilobase of exon per million reads mapped (FPKM). Finally, the obtained gene and their functional annotation after assembly were compared with the protein sequences in the five public databases (Swiss-Prot, NR, KEGG, KOG and Pfam).

### Validation of miRNA expression by qRT-PCR

Expression of six randomly selected miRNAs was detected using qRT-PCR. The primers used are shown in Table 1, and synthesized by Sangon Biotech (Shanghai, China). The cDNA was synthesized using the miRNA 1st strand cDNA synthesis kit with gDNA eraser (Accurate biology, Changsha, China) from 1 μg total RNA according to the manufacturer’s instructions. qRT-PCR was performed at a final volume of 20 μl using *PerfectStart*™ Green qPCR SuperMix Kit (TransGen Biotech, Beijing, China) with the LightCycler 96 (Roche, CHE). The abundance of each selected miRNA was normalized to the level of the U6 small nuclear RNA (snRNA). The ratio change of each miRNA was determined by 2^−ΔΔ*C*T^ method [45]. All reactions were performed in triplicate.

**Table 1:** Primer sequences used for qRT-PCR

Primer name	Primer sequence (5′ ~ 3′)
DRE-MIR-193B-3P-F	CCCGCAAAGTCCCGCTAAA
DRE-MIR-193B-3P-R	CAGTGCAGGGTCCGAGGTAT
SSA-MIR-203A-3P-F	GTGAAATGTTTAGGACCACTTG
SSA-MIR-203A-3P-R	CAGTGCAGGGTCCGAGGTAT
SSA-MIR-1338-3P-F	TCAGGTTCGTCAGCCCATG
SSA-MIR-1338-3P-R	CAGTGCAGGGTCCGAGGTAT
SSA-MIR-92A-3P-F	TATTGCACTTGTCCCGGCCT
SSA-MIR-92A-3P-R	CAGTGCAGGGTCCGAGGTAT
SSA-MIR-205A-5P-F	TTCCTTCATTCCACCGGATC
SSA-MIR-205A-5P-R	CAGTGCAGGGTCCGAGGTAT
ONI-MIR-125A-F	CCTGAGACCCTTAACCTGTG
ONI-MIR-125A-R	CAGTGCAGGGTCCGAGGTAT
U6-F	CTCGCTTCGGCAGCACATATACT
U6-R	ACGCTTCACGAATTTGCGTGTC

**Table 2:** RNA-seq data from each juvenile *O. mykiss* scale sample collected at different time points during salinity acclimation

Sample	Raw reads	Clean reads	Valid data/G	Q30/%	Mapped reads
CG_1	89,627,272	83,437,990	12.52G	98.71	72,429,195 (86.81%)
CG_2	90,637,284	87,198,854	13.08G	98.58	78,893,393 (90.48%)
CG_3	80,356,512	74,812,354	11.22G	98.25	64,591,320 (86.34%)
7D_1	92,595,784	82,794,096	12.42G	98.59	71,541,252 (86.41%)
7D_2	95,578,444	89,211,058	13.38G	98.09	76,755,268 (86.04%)
7D_3	90,741,298	83,933,042	12.59G	97.99	73,039,003 (87.02%)
14D_1	91,322,682	76,219,276	11.43G	98.49	63,555,106 (83.38%)
14D_2	94,608,098	75,339,848	11.30G	98.58	62,339,088 (82.74%)
14D_3	90,570,502	80,280,090	12.04G	98.54	67,911,295 (84.59%)
21D_1	94,544,622	76,698,318	11.50G	98.56	67,600,190 (88.14%)
21D_2	91,295,438	86,198,836	12.93G	97.37	73,723,516 (85.53%)
21D_3	88,674,894	85,859,784	12.88G	97.32	72,851,690 (84.85%)

### Statistical analysis

The statistical analyses were performed in SPSS 20.0. Data are expressed as the mean ± SD format. Student′s *t*-test was selected to evaluate differences between two groups in qRT-PCR. A *P-*value < 0.05 was considered to indicate a significant difference.

## Supplementary Information

**Additional file 1: Table S1.** Analysis of miRNA sequences of juvenile *O. mykiss* scales collected at different time points during salinity acclimation.

**Additional file 2: Table S2.** Detailed information for miRNA identification and prediction.

**Additional file 3: Table S3.** Detailed information for miRNA family identification.

**Additional file 4: Table S4.** Detailed information for differentially expressed (DE) miRNAs (|log_2_FC| > 1 and *p* < 0.05).

**Additional file 5: Table S5.** Detailed information for differentially expressed (DE) miRNA-targeted genes (mRNAs) (|log_2_FC| > 1 and *p* < 0.05).

**Additional file 6: Table S6.** Detailed information for the differentially enriched GO term analysis.

**Additional file 7: Table S7.** Detailed information for the differentially enriched KEGG pathways.

**Additional file 8: Fig. S1.** Length distribution of miRNAs found in juvenile *O. mykiss* scales collected at different time points during salinity acclimation.

**Additional file 9.** Supplemental Materials and Methods.

## Data Availability

All of the raw data have been uploaded to the NCBI database Sequence Read Archive (SRA) and the SRR numbers were from SRR15559621 to SRR15559632. The data can be accessed at https://www.ncbi.nlm.nih.gov/bioproject/PRJNA753226. The miRNA database we refer to can be found at ftp://mirbase.org/pub/mirbase/CURRENT/, the Genome Database can be found at https://www.ncbi.nlm.nih.gov/genome/?term=Oncorhynchus%20mykiss, and the mRNA Database can be found at https://www.ncbi.nlm.nih.gov/genome/?term=Oncorhynchus%20mykiss. All of the datasets referenced in this study can be obtained upon reasonable request to the corresponding authors.

## Abbreviations

ALP: Alkaline phosphatase
TRAcP: Tartrate-resistant acid phosphatase
Ca: Calcium
P: Phosphorus
Ca/P: Molar mass ratio of calcium to phosphorus
ICP-MS: Inductively Coupled Plasma Mass Spectrometry
miRNAs: MicroRNAs
snRNA: Small nuclear RNA
FAO: Food and Agriculture Organization of the United Nations
MS-222: 3-Aminobenzoic acid ethyl ester methanesulfonate
RNA-seq: RNA sequencing
CG: Control group
7D: 7 days
14D: 14 days
21D: 21 days
CST miRNAs: Differentially expressed miRNAs from clusters with significant trends in the cluster analysis
CST mRNAs: Differentially expressed target genes of 22,374 CST miRNA showed a significant trend in cluster analysis
DE miRNAs: Differentially expressed miRNAs
FPKM: Fragments per kilobase of exon per million reads mapped
FRP: Fiber Reinforce Plastic
STEM: Short Time-series Expression Miner
GO: Gene oncology
KEGG: Kyoto encyclopedia of gene and genomes
BP: Biological process
CC: Cellular component
MF: Molecular function
qRT-PCR: Quantitative real-time PCR
