# Supplementary material for: Effects of salinity acclimation on histological characteristics and miRNA expression profiles of scales in juvenile rainbow trout (Oncorhynchus mykiss)
Source: BMC Genomics. 2022 Apr 12;23:300. doi: 10.1186/s12864-022-08531-7 (PMC9006599; doi:10.1186/s12864-022-08531-7)
Supplement: Supplementary file 1 — Additional file 1: Table S1. Analysis of miRNA sequences of juvenile O. mykiss scales collected at different time points during salinity acclimation. [file 12864_2022_8531_MOESM1_ESM.docx]

**Table S1** Analysis of miRNA sequences of juvenile *O. mykiss* scales collected at different time points during salinity acclimation

| Type | CG | | 7D | | 14D | | 21D | |
| --- | --- | --- | --- | --- | --- | --- | --- | --- |
|  | Total (%) | unique (%) | Total (%) | unique (%) | Total (%) | unique (%) | Total (%) | unique (%) |
| Raw reads | 11,277,023  (100.00) | 921,415  (100.00) | 11,052,110  (100.00) | 824,625  (100.00) | 12,969,211  (100.00) | 946,666  (100.00) | 11,929,493  (100.00) | 894,351  (100.00) |
| 3ADT& length filter | 5,251,562  (46.50) | 567,743  (61.85) | 5,157,869  (45.88) | 552,577  (66.90) | 6,958,124  (53.57) | 689,174  (72.84) | 6,294,338  (52.96) | 644,677  (72.06) |
| Junk reads | 30,525  (0.27) | 3,428  (0.37) | 23,126  (0.21) | 2,505  (0.30) | 9,056  (0.07) | 2,208  (0.23) | 8,140  (0.07) | 1,738  (0.19) |
| Rfam | 542,449  (4.83) | 7,238  (0.79) | 564,990  (5.15) | 7,133  (0.88) | 509,731  (3.94) | 8,623  (0.92) | 679,638  (5.69) | 10,551  (1.19) |
| mRNA | 41,515  (0.37) | 1,721  (0.18) | 33,869  (0.32) | 1,167  (0.14) | 46,402  (0.36) | 1,114  (0.12) | 66,780  (0.55) | 1,308  (0.15) |
| Repeats | 109,582  (0.98) | 517  (0.06) | 154,111  (1.37) | 481  (0.06) | 66,809  (0.51) | 787  (0.08) | 70,264  (0.58) | 891  (0.10) |
| valid reads | 5,412,133  (48.04) | 341,328  (36.81) | 5,272,049  (48.45) | 261,252  (31.77) | 5,447,228  (42.07) | 245,627  (25.90) | 4,880,402  (40.72) | 236,173  (26.42) |
