# Supplementary material for: Effects of salinity acclimation on histological characteristics and miRNA expression profiles of scales in juvenile rainbow trout (Oncorhynchus mykiss)
Source: BMC Genomics. 2022 Apr 12;23:300. doi: 10.1186/s12864-022-08531-7 (PMC9006599; doi:10.1186/s12864-022-08531-7)
Supplement: Supplementary file 8 — Additional file 8: Fig. S1. Length distribution of miRNAs found in juvenile O. mykiss scales collected at different time points during salinity acclimation. [file 12864_2022_8531_MOESM8_ESM.docx]

**Fig. S1** Length distribution of miRNAs found in juvenile *O. mykiss* scales collected at different time points during salinity acclimation.
