# Supplementary material for: Effects of salinity acclimation on histological characteristics and miRNA expression profiles of scales in juvenile rainbow trout (Oncorhynchus mykiss)
Source: BMC Genomics. 2022 Apr 12;23:300. doi: 10.1186/s12864-022-08531-7 (PMC9006599; doi:10.1186/s12864-022-08531-7)
Supplement: Supplementary file 9 — Additional file 9. Supplemental Materials and Methods. [file 12864_2022_8531_MOESM9_ESM.docx]

**Quality assurance (QA) and quality control (QC)**

All samples were performed in triplicate, digested and used standard material (GBW10024 (GSB-15), Scallop Biological Component Analysis Standard Material) to check the analysis program. To avoid potential contamination, stainless steel tools were used to collect scale samples and were readily cleaned with ultrapure water. All chemicals used in this study were guaranteed reagents (GR) and purchased from ANPEL Laboratory Technologies Co., Ltd. (Shanghai, China). All glassware was soaked in 20% nitric acid for at least 24h, and was rinsed several times with ultrapure water. Blank and control standards test were performed simultaneously to correct the experimental results.
